# Bacteriophage Cocktail Can Effectively Control *Salmonella* Biofilm in Poultry Housing

**DOI:** 10.3389/fmicb.2022.901770

**Published:** 2022-06-29

**Authors:** Paweł Korzeniowski, Paulina Śliwka, Maciej Kuczkowski, Dušan Mišić, Agata Milcarz, Marta Kuźmińska-Bajor

**Affiliations:** ^1^Department of Biotechnology and Food Microbiology, Faculty of Biotechnology and Food Sciences, Wrocław University of Environmental and Life Sciences, Wrocław, Poland; ^2^Department of Epizootiology and Clinic of Birds and Exotic Animals, Faculty of Veterinary Medicine, Wrocław University of Environmental and Life Sciences, Wrocław, Poland; ^3^Department of Functional Food Products Development, Faculty of Biotechnology and Food Sciences, Wrocław University of Environmental and Life Sciences, Wrocław, Poland

**Keywords:** *Salmonella* Enteritidis, bacteriophage, phage cocktail, biocontrol, biofilm, poultry

## Abstract

*Salmonella enterica* serovar Enteritidis (*S*. Enteritidis) is the major contaminant of poultry products, and its ability to form biofilms on produced food and poultry farm processing surfaces contributes to *Salmonella* transmission to humans. Bacteriophages have come under increasing interest for anti-*Salmonella* biofilm control. In this study, we used the three previously sequenced and described phages UPWr_S1, UPWr_S3, and UPWr_S4 and a phage cocktail, UPWr_S134, containing these three phages to degrade biofilms formed by two *S*. Enteritidis strains, 327 lux and ATCC 13076, *in vitro*. It was found that treatment with bacteriophages significantly reduced biofilm on a 96-well microplate (32–69%) and a stainless steel surface (52–98%) formed by *S*. Enteritidis 327 lux. The reduction of biofilm formed by *S*. Enteritidis ATCC 13076 in the 96-well microplate and on a stainless steel surface for bacteriophage treatment was in the range of 73–87% and 60–97%, respectively. Under laboratory conditions, an experimental model utilizing poultry drinkers artificially contaminated with *S*. Enteritidis 327 lux and treated with UPWr_S134 phage cocktail was applied. In *in vitro* trials, the phage cocktail significantly decreased the number of *Salmonella* on the surface of poultry drinkers. Moreover, the phage cocktail completely eradicated *Salmonella* from the abundant bacterial load on poultry drinkers in an experimentally infected chickens. Therefore, the UPWr_S134 phage cocktail is a promising candidate for *Salmonella* biocontrol at the farm level.

## Introduction

Bacteria from the genus *Salmonella* are among the most common causes of foodborne infections worldwide, and despite global harmonization of the strict and continuous monitoring of microbiological safety of food, millions of people are infected every year. According to the European Food Safety Authority (EFSA), in 2019, 87,923 confirmed cases of salmonellosis, with the most frequently isolated *Salmonella enterica* serovar Enteritidis (*S*. Enteritidis), were recorded in European countries alone, the source of which was food (The European Union One Health 2019 Zoonoses Report, 2021).

Due to market demands, there has been an increase in the volume of intensive poultry farming in recent years. As regards the entry of this pathogen into the food chain, the presence and maintenance of *Salmonella* in poultry flocks is of particular importance, so that poultry products, meat, and eggs are traditionally the main sources of infection for humans (Sanchez et al., 2002; Foley et al., 2011). In the vast majority of European countries, *Salmonella* is detected in ~1–2% of poultry flocks, with the most frequently isolated *Salmonella enterica* serovar Enteritidis predominating in breeder (24.8%) and laying hen (43.7%) sectors and being the third most common (8.8%) in broilers, confirming the importance of this serovar (Koutsoumanis et al., 2019; Sevilla-Navarro et al., 2020).

It is commonly thought that *Salmonella* enters poultry farms *via* biological vectors, insects, and rodents. However, the link between the survival of *Salmonella* on abiotic surfaces and the occurrence of salmonellosis in chickens has been clearly demonstrated (Crump et al., 2002; Jones, 2011). The survival of *Salmonella* on abiotic surfaces is one part of the life cycle of this bacterial species and is related to its multicellular organization. The mechanisms that *Salmonella* activates for survival in the environment are different from the factors responsible for the pathogenesis of the disease. *Salmonella* can also reach poultry farms with contaminated water, animal feed, and raw materials of animal and plant origin, which are also related to their ability to invade plant tissues and to survive on abiotic surfaces in facilities where these raw materials were processed (Bailey, 1988; Nissen et al., 2001; Chia et al., 2009; Fatica and Schneider, 2011). The presence of *Salmonella* has been detected in mills for chopping and mixing food, on conveyor belts, packaging machines, storage places, and other surfaces with which animal feed comes into contact. Thus, it is estimated that equipment and all surfaces on poultry farms and in animal feed production sites made of plastic, stainless steel, wood, and glass can be a substrate for the development of *Salmonella* biofilms (Nesse et al., 2003; Vestby et al., 2009). Biofilms are extremely difficult to prevent and eradicate, because, in biofilms, bacteria show increased resistance to various stressors (drying, antibiotic action, disinfectants, heavy metal ions, and UV radiation). Consequently, modern strategies to combat pathogenic bacteria are insufficient to control pathogens organized into biofilms (Maciorowski et al., 2006; Burmølle et al., 2010).

It has long been hypothesized that the number of *Salmonella in vitro* or the organism of infected humans and animals can be reduced or even eliminated with the use of bacteriophages. Bacteriophages were discovered almost 100 years ago, and since then numerous papers have been published about their application against *Salmonella* (Wei et al., 2019; Zbikowska et al., 2020). In poultry, attempts have been made to combat this pathogen *in vivo*, with variable results, including complete elimination of *S*. Enteritidis (Atterbury et al., 2007) as well as no influence on the number of pathogens (Borie et al., 2009). It can be seen that in most of the reported experiments, the chickens were already infected with *Salmonella* (artificial infection) and phages were administered directly to the crop (Andreatti Filho et al., 2007; Bardina et al., 2012) or *via* drinking water (Clavijo et al., 2019; Vaz et al., 2020), aerosol spray (Borie et al., 2008, 2009), and food (Lim et al., 2012; Adhikari et al., 2017) in an attempt to reduce the number of *Salmonella in vivo*. Based on our knowledge of the published data, no experiments have been conducted to test the hypothesis that *Salmonella* is present on abiotic surfaces such as poultry drinkers and to attempt their eradication. We established a new research model useful in phage ability to eradicate *Salmonella* from poultry drinkers as the main point in pathogen horizontal transmission within a poultry flock by applying phages *in vivo* with an engineered *S*. Enteritidis strain. Further, in this study, we report for the first time the UPWr_S134 phage cocktail anti-biofilm activity in an experimental chicken model. The anti-biofilm activity of single phages UPWr_S1, UPWr_S3, and UPWr_S4 and phage cocktail UPWr_S134 was also indicated in 96-well microtiter plates and contaminated stainless steel washers in *in-vitro* models.

## Materials and Methods

### Bacterial Strains

*Salmonella* Enteritidis (*S*. Enteritidis) strains termed 327 lux, A41 and A36 from the Strain Collection of the Department of Epizootiology and Clinic of Bird and Exotic Animals, Wrocław University of Environmental and Life Sciences, isolated from diseased chickens, were used in this study. *S*. Enteritidis 327 lux was genetically modified for bioluminescence and resistance to erythromycin as previously described by Riedel et al. (2007), and it has already been used in previous investigations by Kuzmińska-Bajor et al. (2015). For this purpose, the chromosomal integration vector p16Slux containing the *lux* operon from *Photorhabdus luminescens* and consisting of five genes, *luxCDABE*, was used. The *S*. Enteritidis 327 lux strain exhibits a high ability to cause salmonellosis in chickens and contains markers such as erythromycin resistance and light production, which apparently allows this strain to be easily distinguished from chicken microflora. The biofilm production ability of *S*. Enteritidis 327 lux was previously tested according to Stepanovic et al. (2004), and it was categorized as a moderate biofilm producer. *S*. Enteritidis ATCC 13076 (Microbiologics, St. Cloud, MN, USA), which was categorized as a strong biofilm producer, was used as a control in this study.

### Phage Origin, Propagation, and Titration

The bacteriophages used in this study, termed UPWr_S1, UPWr_S3, and UPWr_S4 were previously sequenced and described (Kuzmińska-Bajor et al., 2021). UPWr_S1, UPWr_S3, and UPWr_S4 phages exhibit a lytic life cycle and are deprived of the ability to promote lysogeny, toxin production, antibiotic resistance, or virulence.

*S*. Enteritidis A41 was used as host for *in vitro* amplification of the phage UPWr_S1, whereas *S*. Enteritidis A36 was used as a host for UPWr_S3 and UPWr_S4. Phages were propagated as previously described by Oliveira (2009). Briefly, 10 ml of LB broth (A&A Biotechnology, Poland) was inoculated with a single respective host *Salmonella* colony following overnight incubation at 37°C with shaking at 150 rpm. Ten milliliters of fresh sterile LB broth was inoculated with 0.5 ml of previously prepared broth culture and incubated at 37°C and 150 rpm until optical density (OD_600nm_) reached 0.2, which is ~2 × 10^8^ CFU/ml. Then, 5 ml of phage lysate was added and further incubated at 37°C with shaking. In the next step, the bacterial cultures were centrifuged for 10 min at 5,000 × *g* and the supernatant was filtered through 0.22 μm pore size syringe filters (Merck Millipore, USA). The obtained phage lysate was added to 150 ml of refreshed host culture (OD_600nm_ = 0.2) host culture and incubated overnight at 37°C. Then, the centrifugation and filtration steps were repeated. Bacteriophage preparation titer was determined using the routine test dilution method (Adams, 1959). Phages UPWr_S1, UPWr_S3, and UPWr_S4 were mixed into a phage cocktail named UPWr_S134. Phage cocktail and phages alone were analyzed for their ability to reduce the number of *S*. Enteritidis in *in-vitro* and *in-vivo* studies.

### Investigation of Biofilm Reduction With Bacteriophages in 96-Well Microplate Assay

The modified method proposed by Woodward et al. (2000) was applied to quantitatively determine the phage efficacy in *Salmonella* biofilm reduction. For this purpose, overnight *S*. Enteritidis 327 lux and *S*. Enteritidis ATCC 13076 cultures were diluted in LB broth to match the optical density (OD_600nm_) 0.2 (~2 × 10^8^ CFU/ml) and 200 μl of this suspension was transferred to each well of 96-well plates (Sarstedt, Germany). Microtiter plates were incubated at 37°C for 72 h. Wells were washed twice with sterile phosphate saline buffer (PBS; Sigma-Aldrich, Germany), to remove planktonic cells. For biofilm degradation, UPWr_S1, UPWr_S3, and UPWr_S4 and the phage cocktail UPWr_S134 were used. The volume of 200 μl of bacteriophage lysates was added to each well at final titers of 10^4^, 10^5^, 10^6^, 10^7^, 10^8^, and 10^9^ PFU/ml. Plates were then incubated at 37°C for 4 h, rinsed with PBS three times, and allowed to air-dry. The remaining biofilms were quantified by staining with 0.5% crystal violet (Merck, Germany) for 20 min followed by elution two times with PBS. Crystal violet was dissolved using 96% ethanol (Sigma-Aldrich, Germany). The absorbance of the released color was measured using an automated microtiter plate reader (Spark Tecan, Switzerland) at 570 nm. Biofilms not incubated with bacteriophages were used as positive controls. All experiments were performed in triplicate.

### Effectiveness of Bacteriophages in Reducing *S*. Enteritidis on the Stainless Steel Surface

To quantitatively determine the phage efficacy against *Salmonella* biofilm formed on a stainless steel surface, the modified method proposed by Orsinger-Jacobsen et al. (2013) was applied. Overnight *S*. Enteritidis 327 lux and *S*. Enteritidis ATCC 13076 cultures were suspended in 250 ml Erlenmeyer flasks with 100 ml of LB containing sterile 10 mm diameter stainless steel washers to match the optical density (OD_600nm_) of 0.2 (~2 × 10^8^ CFU/ml). The flasks were then incubated for 72 h at 37°C on a rotary shaker set at 150 rpm. In the next step, each washer was transferred using sterile forceps to a well of a 24-well plate (Sarstedt, Germany) containing different phage titers (10^4^, 10^5^, 10^6^, 10^7^, 10^8^, and 10^9^ PFU/ml) of UPWr_S1, UPWr_S3, and UPWr_S4 and the phage cocktail UPWr_S134 or LB as a control and further incubated for 4 h at 37°C. The phage lysates were then removed, steel washers were rinsed with PBS three times to remove non-adherent bacteria, air dried, and the remaining bacteria were measured by crystal violet staining for 20 min followed by elution with PBS twice. In the next step, the washers were transferred to clean 24-well plates containing 96% ethanol to dissolve the crystal violet and incubated for 15 min at room temperature. Finally, steel washers were removed from wells and the absorbance of released color was measured using an automated microtiter plate reader at 570 nm.

### Phage Treatment of *Salmonella* Attached to Poultry Drinker Surface Under Laboratory Conditions

The ability of bacteriophages to reduce the number of *Salmonella in vitro* was examined in artificially contaminated poultry bell drinkers of two parts: a base and a pot with a clip-on type connection (Polmark, Poland). For this purpose, the experiment was performed in triplicate with three drinker's bases at the same time for one environment, providing three distinct conditions differing in nutrient content (water, LB medium, and LB medium diluted 10 times with sterile water). To fill drinker's base completely, 100 ml of water, LB medium, and diluted LB medium were inoculated with 10^5^ CFU/ml of *S*. Enteritidis 327 lux and mixed with 10^7^ PFU/ml of phage cocktail UPWr_S134. Drinkers were left at 25°C for 9 days. To determine the number of *Salmonella* attached to the drinker surface, swabs were taken daily starting from the 1st day after inoculation. In the swabbing technique, a sterile aluminum foil template containing a 1 × 1 cm square opening (1 cm^2^) was placed on the wall of the poultry drinker. A sterile wooden swab (Matoset Instrument, Poland) was used over the exposed area in all directions. Swab samples were placed in tubes containing 1 ml of sterile PBS and shaken for 30 min at 200 rpm. A series of dilutions of samples were prepared and plated onto LB agar with 500 μg/ml erythromycin (Sigma-Aldrich, Germany). Readings of the results were performed after 24 h of incubation at 37°C, and the obtained results were expressed as *Salmonella* CFU/cm^2^.

### *Salmonella* Eradication From the Poultry Drinker Surface in the Experimental Chicken Model

To determine the effect of the UPWr_S134 phage cocktail on artificially contaminated poultry drinkers, an experimental chicken model was used. The animal experiments were performed according to the International Animal Care Convention and were approved by the Local Ethics Committee for Animal Experimentation (protocol code 114/2015; Wrocław, Poland). Fourteen day-of-hatch healthy broiler chickens were obtained from a local farm and randomly divided into four groups of 10 birds. Each group was placed in wired cages with one drinker per two birds, provided water and feed *ad libitum*, and maintained at an age-appropriate temperature (25°C) for the duration of the whole experiment. Poultry drinkers, containing bases and pots, of experimental group 1 (positive control) were contaminated with *S*. Enteritidis 327 lux with drinking water to maintain the *S*. Enteritidis concentration of 1 × 10^5^ CFU/ml. Every two days, 2 l of fresh water containing 1 × 10^5^ CFU/ml *S*. Enteritidis 327 lux (fresh culture) was added to the pot of each drinker without cleaning the base of the drinker. Bacteriophage cocktail UPWr_S134 was added to water in drinkers of experimental group 2 (negative control) to maintain its final concentration of 1 × 10^7^ PFU/ml of each phage in the cocktail. In this group, every 48 h, 2 l of fresh water containing the appropriate phage cocktail concentration was added. Experimental group 3 chickens drank water inoculated with 1 × 10^5^ CFU/ml *S*. Enteritidis 327 lux and 3 × 10^7^ PFU/ml phage cocktail UPWr_S134. The water with bacteria and phages was changed every 2 days. Group 4 remained untreated. Chicken groups are presented in Table 1. Within 9 days of the experiment, the total number of attached *Salmonella* on the walls of the drinkers was calculated. To determine the number of *Salmonella* as well as the total viable count (TVC), both expressed as CFU/cm^2^, swabs were taken daily from 1 cm^2^ of the surface of five individual drinkers, and bacterial counts were established using the same methodology as for the *in vitro* trials on the poultry drinker model. The number of *S*. Enteritidis 327 lux was estimated by plating a serial dilution onto LB containing 500 μg/ml erythromycin and incubating at 37°C for 24 h. Light-producing colonies were counted using the NightOwl 983 imaging system (Berthold, Germany) which eliminates the risk of counting bacteria other than *S*. Enteritidis 327 lux. To determine the TVC, serial dilutions were plated onto Plate Count Agar (PCA) (Sigma-Aldrich, Germany), incubated at 37°C for 24 h, and the number of colonies was calculated. After 9 days, all the birds were euthanized under anesthesia, and internal organs such as the liver, the spleen, and the bursa of Fabricius were dissected and weighed. Livers were placed in tubes containing 50 ml of sterile PBS and homogenized for 5 min using BagMixer 400 S (Interscience, France), whereas the spleens and the bursas of Fabricius were homogenized in 5 ml of sterile PBS, for 5 min at 20 Hz using Qiagen TissueLyser II (Qiagen, Germany). Serial dilutions were prepared and plated onto LB containing 500 μg/ml erythromycin to determine *S*. Enteritidis 327 lux load in internal organs.

**Table 1:** Chicken experimental design. UPWr_S134 phage cocktail and *S*. Enteritidis 327 lux were constantly administered *via* drinking water.

**Group**	**No. of chickens**	**No. of drinkers**	**Concentration**		**Water change schedule [days]**
			**UPWr_S134**	***S***. **Enteritidis 327 lux**	
1	10	5	-	1 × 10^5^ CFU/ml	2, 4, 6, 8
2	10	5	1 × 10^7^ PFU/ml	-	2, 4, 6, 8
3^a^	10	5	3 × 10^7^ PFU/ml	1 × 10^5^ CFU/ml	2, 4, 6, 8
4	10	5	-	-	2, 4, 6, 8

*1 - S. Enteritidis 327 lux treated positive control*.

*2 - UPWr_S134 treated negative control*.

*3 - Inoculated with S. Enteritidis 327 lux and UPWr/S134 treated*.

*4 - Untreated control*.

a *UPWr_S134 phage cocktail was administered together with S. Enteritidis 327 lux inoculation*.

### Statistical Analysis

Data were collected and analyzed using Statistica version 13 software (TIBCO Software Inc.). The significance level of *p* < 0.05 was predetermined for all statistical tests. The 96-well biofilm assays and *in vitro* phage treatment of *Salmonella* attached to the stainless steel washer and poultry drinker surface were performed in triplicate independently. Bacteria counts were logarithmically transformed prior to statistical analysis. Data obtained from the microplate biofilm assay were analyzed by applying a one-way analysis of variance (ANOVA) along with the least significant difference (LSD) *post-hoc* test. The student's *t*-test was employed for statistical analysis of bacterial reduction in the *in-vitro* and *in-vivo* poultry drinker models. The nonparametric Mann-Whitney *U*-test was carried out to compare readings of bacterial reduction in an experimental chicken model and to compare data collected from estimating bacterial load in chickens' internal organs.

## Results

### Phage Activity Against *Salmonella* Biofilms *in vitro*

**Figure 1:**
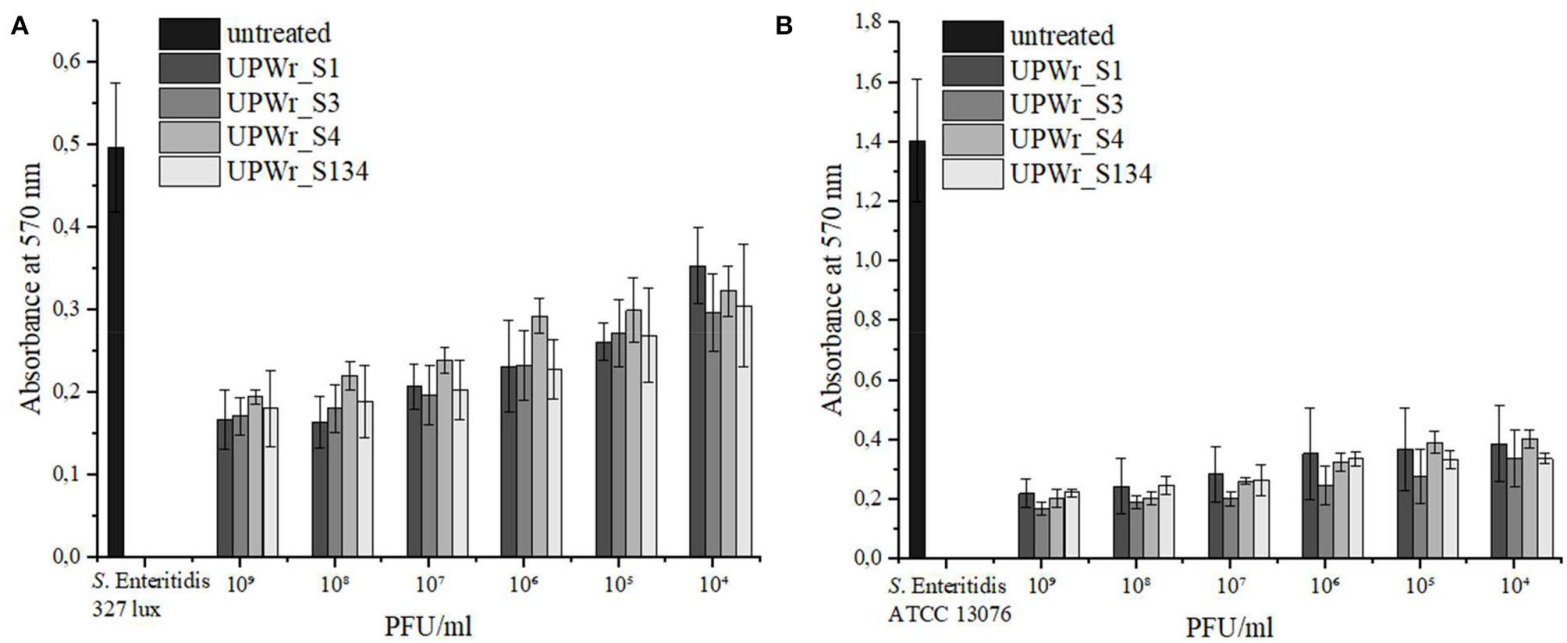
Effect of UPWr_S1, UPWr_S3, and UPWr_S4 phages and phage cocktail UPWr_S134 on biofilm in 96-well microplate. **(A)** Effect of UPWr_S1, UPWr_S3, and UPWr_S4 phages and phage cocktail UPWr_S134 on reduction of biofilm formed by *S*. Enteritidis 327 lux, and **(B)** Effect of UPWr_S1, UPWr_S3, and UPWr_S4 phages and phage cocktail UPWr_S134 on reduction of biofilm formed by *S*. Enteritidis ATCC 13076. Values represent the mean with a standard deviation of three replicates.

The activity of phages UPWr_S1, UPWr_S3, and UPWr_S4 and the phage cocktail UPWr_S134 in biofilm degradation was determined by crystal violet staining in 96-well plates. The *S*. Enteritidis 327 lux and ATCC 13076 strains were classified as moderate and strong biofilm producers, respectively, according to the classification suggested by Stepanovic et al. (2004). The control strain *S*. Enteritidis ATCC 13076 was found to have ~2.8 times greater ability to form a biofilm than *S*. Enteritidis 327 lux and exhibited higher sensitivity to treatment with UPWr_S1, UPWr_S3, and UPWr_S4 phages alone and the phage cocktail UPWr_S134 (Figure 1). Biofilm biomass degradation compared to the control observed for all tested phage titers of single-phage lysates and the UPWr_S134 cocktail against *S*. Enteritidis ATCC 13076 was highly significant (*p* < 0.01) (Supplementary Tables 1, 3). For the highest phage titer 10^9^ PFU/ml, the reduction level of biofilm produced by *S*. Enteritidis 327 lux did not differ between treatments and was estimated to be 69, 63, 65, and 65% for phages UPWr_S1, UPWr_S3, and UPWr_S4 and the phage cocktail UPWr_S134, respectively. For treatment with the lowest titer of 10^4^ PFU/ml, degradation was estimated to be 32, 41, 34, and 37% for phages UPWr_S1, UPWr_S3, and UPWr_S4 and the phage cocktail UPWr_S134, respectively, and did not differ between treatments (Figure 1A). Treatment of *S*. Enteritidis ATCC 13076 biofilm in a 96-well microtiter plate with the titer of 10^9^ PFU/ml of phages UPWr_S1, UPWr_S3, and UPWr_S4 and the phage cocktail UPWr_S134 resulted in a drastic decrease of biofilm biomass of 84, 87, 82, and 83%, respectively. Applying single phages and the phage cocktail at a low titer of 10^4^ PFU/ml affected efficient biofilm removal activity, which was still great and estimated to be 72, 75, 65, and 76% for UPWr_S1, UPWr_S3, and UPWr_S4 and the phage cocktail UPWr_S134, respectively (Figure 1B). No significant differences were found for *S*. Enteritidis 327 lux and ATCC 13076 biofilm treatment with phages alone and in a mixture for all tested titers of 10^4^-10^9^ PFU/ml (*p* > 0.05). The dose-dependent effect observed was only for the phage UPWr_S4 in the degradation of biofilm formed by these two *S*. Enteritidis strains (Supplementary Tables 2, 4).

### Degradation of *S*. Enteritidis Biofilm on Stainless Steel

**Figure 2:**
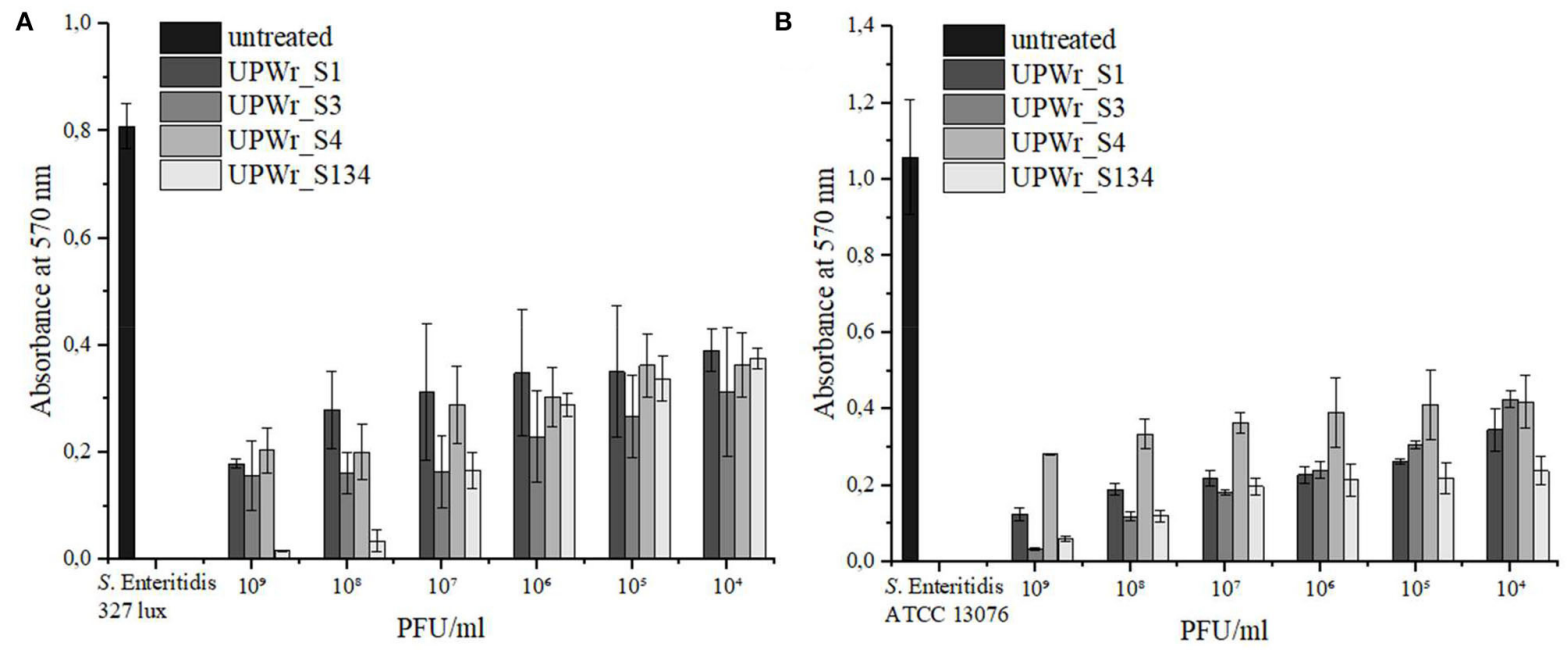
Effect of UPWr_S1, UPWr_S3, and UPWr_S4 phages and phage cocktail UPWr_S134 on biofilm on stainless steel washers. **(A)** Effect of UPWr_S1, UPWr_S3, and UPWr_S4 phages and phage cocktail UPWr_S134 on reduction of biofilm formed by *S*. Enteritidis 327 lux, and **(B)** effect of UPWr_S1, UPWr_S3, and UPWr_S4 phages and phage cocktail UPWr_S134 on reduction of biofilm formed by *S*. Enteritidis ATCC 13076. Values represent the mean with a standard deviation of three replicates.

Stainless steel washers used in this study were evaluated for phage lytic activity against strains of *S*. Enteritidis that are able to produce biofilm. The strong biofilm producer *S*. Enteritidis ATCC 13076 showed ~1.4 times higher ability to form biofilm on a stainless steel surface than *S*. Enteritidis 327 lux, and biofilms produced by each strain were effectively destroyed by monophage lysates and the phage cocktail (*p* < 0.01; Figure 2). Statistically significant biofilm removal compared to the control was observed for all tested titers of UPWr_S1, UPWr_S3, and UPWr_S4 phages and the UPWr_S134 phage cocktail against both *S*. Enteritidis strains (*p* < 0.01) (Supplementary Tables 5, 7). A dose-dependent effect of reduction of pre-formed *S*. Enteritidis 327 lux biofilm was observed for the UPWr_S134 phage cocktail between titers 10^5^ and 10^8^ PFU/ml. For this strain, the UPWr_S134 phage cocktail exhibited greater efficiency in biofilm degradation for high titers of 10^8^ and 10^9^ PFU/ml (96 and 98%) in comparison to monophage lysates (*p* < 0.01). In total, 54% of biofilm removal for a titer of 10^4^ PFU/ml of the UPWr_S134 phage cocktail was observed. A low titer of 10^4^ PFU/ml of single phages UPWr_S1, UPWr_S3, and UPWr_S4 showed the ability to lyse biofilm-based *S*. Enteritidis 327 lux at levels of 52, 61, and 55%, respectively, whereas these phages at the highest titer of 10^9^ PFU/ml caused biofilm degradation estimated to be 78, 81, and 75%, respectively. No significant differences were found for treatments against *S*. Enteritidis 327 lux with phages alone and in a mixture for titers between 10^4^ and 10^7^ PFU/ml (*p* > 0.05) (Figure 2A, Supplementary Table 7). In the case of strain, *S*. Enteritidis ATCC 13076, UPWr_S1, UPWr_S3, and UPWr_S4 phages and the UPWr_S134 phage cocktail treatments with the highest titer of 10^9^ PFU/ml reached effectiveness up to 88, 97, 73, and 94%, respectively (Figure 2B). The high effectiveness to reduce *S*. Enteritidis ATCC 13076 of 67, 60, 60, and 77% was observed for the low titer of 10^4^ PFU/ml of phages UPWr_S1, UPWr_S3, and UPWr_S4 and the UPWr_S134 phage cocktail, respectively. A dose-dependent effect was found for the phage UPWr_S3. Phage UPWr_S4 exhibited significantly lower activity in the elimination of *S*. Enteritidis ATCC 13076 cells from biofilm structure for titers of 10^5^-10^9^ PFU/ml in comparison to other treatments (Supplementary Table 8). It was observed that the phage cocktail UPWr_S134 was significantly more effective in reducing biofilm formed by *S*. Enteritidis ATCC 13076 for low titers of 10^4^ and 10^5^ PFU/ml in comparison to monophage lysates (*p* < 0.01).

### Phage Treatment of *S*. Enteritidis 327 Lux on Poultry Drinker Surface

**Figure 3:**
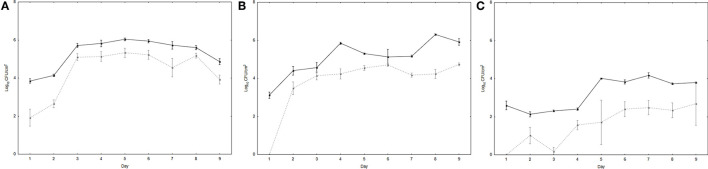
Effectiveness of phage cocktail UPWr_S134 in reduction of *S*. Enteritidis 327 lux on poultry drinker surface. Effect of phage cocktail UPWr_S134 on the number of *S*. Enteritidis 327 lux in the LB **(A)** and LB diluted 10-fold **(B)** and water **(C)**. Values represent the mean with a standard deviation of three determinations. Black lines indicate the number of *S*. Enteritidis 327 lux on the drinker surface and gray dashed lines indicate the number of *S*. Enteritidis 327 lux on the drinker surface treated with phage cocktail UPWr_S134.

UPWr_S134 phage cocktail's ability to reduce the number of *Salmonella* attached to the surface of poultry drinkers was tested *in vitro* in a mixture containing both phages and bacteria. This model reflects conditions on a farm where drinking water with phage additives could be contaminated by infected chickens *via* the fecal-oral route. Differences between controls inoculated only with bacteria and drinkers treated with phages were observed for all three environments (*p* < 0.01; Figure 3). The number of *S*. Enteritidis 327 lux was significantly higher on the surface of the drinkers with diluted and undiluted LB medium in comparison to water. In LB medium, the number of *S*. Enteritidis 327 lux reached the level of ~6 log_10_ CFU/cm^2^ starting on day 3 (Figure 3A), and in 10-fold diluted LB medium, it was estimated to be ~5–6 log_10_ CFU/cm^2^ from day 2 (Figure 3B). No significant results were found between these two media in bacterial load (*p* > 0.05). In drinkers with a water environment, the number of bacteria isolated from the surface was significantly lower than in diluted and undiluted LB medium and reached 4 log_10_ CFU/cm^2^ at post-infection day 5 (*p* < 0.01) (Figure 3C). For all three *Salmonella*-contaminated environments, the greatest differences were displayed on days 1 and 2 between drinkers treated and not treated with the UPWr_S134 phage cocktail. In drinkers with 10-fold diluted LB medium, no viable *Salmonella* cells were detected on day 1. In water, the difference between control drinkers and drinkers with *Salmonella* and phages was the largest and was calculated to be ~2 log_10_ (*p* < 0.01), whereas in 10-fold diluted LB and LB medium, the number of *S*. Enteritidis 327 lux on the drinker surface was reduced by nearly 1 log_10_ starting on day 3.

### UPWr_S134 Phages Effectively Reduced the Number of *Salmonella* From the Poultry Drinker Surface in the Experimental Chicken Model

**Figure 4:**
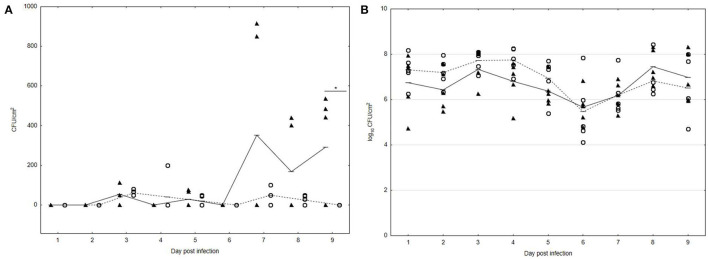
Effect of phage cocktail UPWr_S134 on bacteria attached to the poultry drinker surface in an experimental chicken model. **(A)** Effect of phage cocktail UPWr_S134 on the number of *S*. Enteritidis 327 lux. **(B)** Effect of phage cocktail UPWr_S134 on the total viable count. Results for bacterial load show counts for individual drinkers plus the mean (*n* = 5 per group). *p < 0.05 indicates a significant difference between control and treatment groups. Black triangles indicate control group 1 infected with *S*. Enteritidis 327 lux and empty circles indicate group 3 treated with both *S*. Enteritidis 327 lux and phage cocktail UPWr_S134.

For evaluation of the UPWr_S134 phage cocktail's ability to eliminate *S*. Enteritidis 327 lux from the multispecies community formed on the poultry drinker surface, analysis with an experimental chicken model was performed. In drinkers from all experimental groups, a high number of TVC was detected. The TVC on the poultry drinker surface did not change significantly during the whole experimental period, with the range of 5–8 log_10_ CFU/cm^2^ between the untreated group 1 (black lines with black triangles) and group 3 treated with the UPWr_S134 phage cocktail (dashed lines with empty circles), respectively (*p* < 0.05) (Figure 4B). Treatment of poultry drinkers filled with drinking water contaminated with *Salmonella* and the phage cocktail UPWr_S134 resulted in inhibition of *S*. Enteritidis 327 lux multiplication, contrary to phage-untreated drinkers (*p* < 0.01). In drinkers from group 3 treated with UPWr_S134 phages, the mean number of *Salmonella* did not exceed 10^2^ CFU/cm^2^ during the experiment and on the last day (9 days after infection) *S*. Enteritidis 327 lux were not detected in swabs (empty circles) (Figure 4A). In drinkers from group 1 contaminated with *Salmonella* and not treated with phages, the detected number of *Salmonella* starting on day 7 was higher than in group 3 and ranged between 1.5 and 3.5 × 10^2^ CFU/cm^2^ (*p* < 0.05; black triangles).

**Figure 5:**
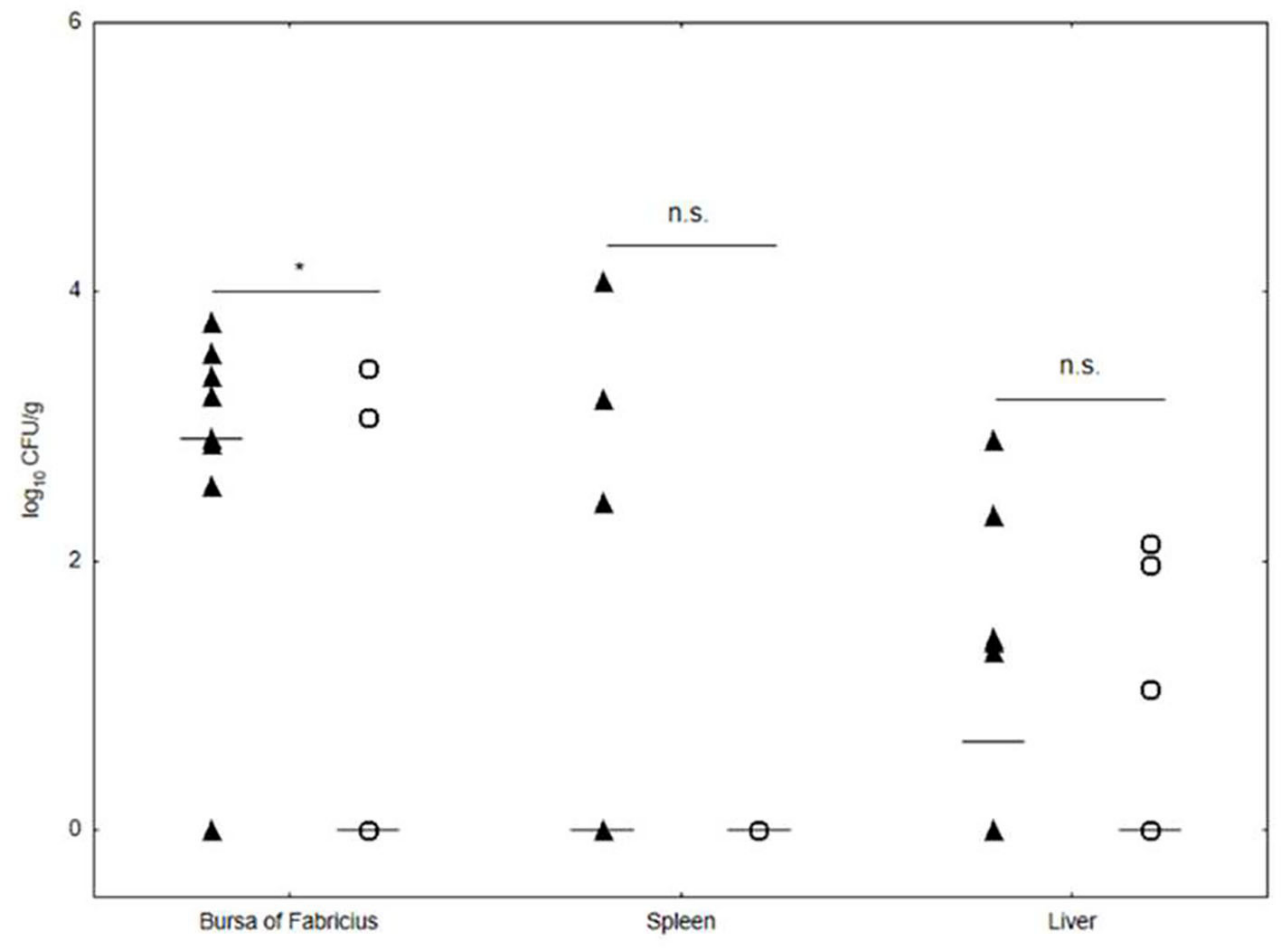
Effect of phage cocktail UPWr_S134 on *S*. Enteritidis 327 lux number in internal organs in the experimental chicken model. Results for bacterial load are shown as counts for individual animals plus the median (*n* = 10 per group). n.s., not significant, *p < 0.05 indicates a significant difference between control group 1 infected (black triangle) with *S*. Enteritidis 327 lux and the group 3 (empty circle) infected with *S*. Enteritidis 327 lux treated with phage cocktail UPWr_S134.

In addition, the number of *S*. Enteritidis 327 lux in chicken organs was estimated. All chicks in groups 1 and 3 showed no clinical signs of salmonellosis and no postmortem findings. The negative control group 2 and uninfected and untreated group 4 remained *Salmonella* free during the experiment. In group 1, the number of lymphoid organ bursa of Fabricius colonized by *S*. Enteritidis 327 lux was 8/10 (80%) with the mean load of 3.2 ± 0.4 log_10_ CFU/g, which was significantly higher in comparison to the phage-treated group 3 with 2/10 (20%) infected organs with ~2.6 ± 0.26 log_10_ CFU/g (*p* < 0.05) (Figure 5). The incidence of *S*. Enteritidis 327 lux-positive organs such as the liver and the spleen did not differ significantly as compared with the positive control group 1. However, in samples from the *Salmonella*-infected control group 1, the number of organs with a viable bacterial count was higher than in the phage-treated group. In samples of the liver from group 1, the number of colonized organs was 5/10 (50%) with 2.0 ± 0.71 log_10_ CFU/g (black triangles). The number of *S*. Enteritidis 327 lux-positive liver samples of the phage-treated group 3 was 3/10 (30%) with an average of 1.4 ± 0.6 log_10_ CFU/g (empty circles). In 3/10 spleen samples, *Salmonella* was detected with the mean estimated to be 3.2 ± 0.82 log_10_ CFU/g (black triangles). No viable *S*. Enteritidis 327 lux cells were detected in spleen samples collected from chicken infected with *Salmonella* and treated with phage cocktail UPWr_S134 (empty circles).

## Discussion

*Salmonella* can be disseminated to poultry flocks through several sources such as drinking water, feed, equipment, and wildlife. Since *Salmonella* occurs in the flock, it can be easily transmitted between birds *via* the fecal-oral route with water and drinkers as the main points of horizontal transmission. Water contaminated with feces containing organic material and intestinal microflora contributes to the development of multispecies bacterial communities on the poultry drinker surface and may contain pathogens such as *Salmonella* (Maes et al., 2019). There is a striking paucity of data on the impact of phages on the biofilm developed in the poultry farm environment. The interest in *Salmonella* control in the poultry industry leads to renewed consideration of the use of bacteriophages, which has been recently reviewed by Zbikowska et al. (2020). Since biofilms act as important environmental reservoirs for *Salmonella*, phages have been considered as an effective tool in inhibiting the formation and eradication of its biofilm developed under laboratory conditions on surfaces constituting part of the equipment elements commonly found in the poultry industry such as plastic and stainless steel (Gutiérrez et al., 2016).

UPWr_S1, UPWr_S3, and UPWr_S4 phages are capable of infecting zoonotic pathogens such as *S*. Enteritidis as well as *S*. Gallinarum, which is the etiological factor of fowl typhoid. Regardless of their ability to infect various *Salmonella* serovars and lytic life cycle, they can be considered useful tools in the biological control of salmonellosis (Kuzmińska-Bajor et al., 2021). In this study, we found that UPWr_S1, UPWr_S3, and UPWr_S4 phages alone and the UPWr_S134 phage cocktail exhibited a great ability to decrease the number of *Salmonella* from biofilm structures in a 96-well microtiter plate and on stainless steel washers formed by strong and moderate biofilm producers, *S*. Enteritidis ATCC 13076 and 372 lux strains, respectively. A previous study on *S*. Typhimurium and *S*. Enteritidis showed significant biofilm eradication by phage cocktail at titers of 7 log_10_ and 8 log_10_ PFU/mL in a 96-well microplate with the reduction levels varying between 44 and 63% (Gong and Jiang, 2017; Islam et al., 2019; Esmael et al., 2021). The ability of UPWr_S1, UPWr_S3, and UPWr_S4 phages and the UPWr_S134 phage cocktail to eliminate bacteria from biofilm formed by *S*. Enteritidis 372 lux corresponds to the average reduction level of most *Salmonella*-targeting phage cocktails, whereas monophage and phage cocktail treatment of biofilm formed by the strong biofilm producer *S*. Enteritidis ATCC 13076 resulted in almost complete biofilm eradication. *S*. Enteritidis ATCC 13076 was used previously for mixed biofilm production with the strain *S*. Typhimurium ATCC 14028 and the reduced levels of 44.28 and 58.14% were recorded for titers of 7 log_10_ and 8 log_10_ PFU/ml, respectively, of phage cocktail containing the phages LPSTLL, LPST94, and LPST153 (Islam et al., 2019). The authors reported a low impact of *Salmonella* strains' synergistic effect on biofilm behavior. Moreover, the elimination of bacteria from the biofilm structure by UPWr_S1, UPWr_S3, and UPWr_S4 phages and the UPWr_S134 phage cocktail was substantially more effective than that shown by LPSTLL, LPST94, and LPST153 in the 96-well microplate and on stainless steel surfaces (Islam et al., 2019). Previously, it was found that phage treatment displayed variable effects on the elimination of *Salmonella* from biofilm structure and showed considerable variation between different phages and *Salmonella* serotypes, including effective biofilm removal (Karaca et al., 2015; Milho et al., 2018; Yüksel et al., 2018) and no effect in biofilm dispersion (Gong and Jiang, 2017; de Ornellas Dutka Garcia et al., 2017). Effective reduction of the number of *S*. Enteritidis ATCC 13076 and *S*. Enteritidis 372 lux in a 96-well microtiter plate and from a stainless steel surface shown by UPWr_S1, UPWr_S3, and UPWr_S4 phages and the UPWr_S134 phage cocktail confirms their great efficacy against *Salmonella* biofilms for phages used individually and when combined in a phage cocktail. Commonly, phages considered tools for biocontrol are mixed into a cocktail. Phage cocktails are applied to broaden single phages' host range or increase antibacterial activity (Gill and Hyman, 2010; Kutateladze and Adamia, 2010; Lu and Koeris, 2011; Chan and Abedon, 2012). It should be pointed out that host range is not a stable property (Hyman and Abedon, 2010; Koskella and Meaden, 2013; Ross et al., 2016) and phages can evolve to develop phage resistance or overcome this resistance (Stern and Sorek, 2011; Samson et al., 2013; Chaturongakul and Ounjai, 2014). This has many implications for phage biology as well as for their practical applications. Although UPWr_S1, UPWr_S3, and UPWr_S4 phages showed similar biofilm removal properties in comparison to the UPWr_S134 phage cocktail and they exhibited a similar lytic profile (Kuzmińska-Bajor et al., 2021), for studies on the potential anti-biofilm application in a poultry watering system, the UPWr_S134 phage cocktail was applied.

Data concerning phage-dependent biofilm reduction on plastic surfaces in the poultry industry have been less frequently reported. de Ornellas Dutka Garcia et al. (2017) observed that *Salmonella* spp. biofilm formed on plastic surfaces was more susceptible to phage treatment in comparison to biofilms formed on glass and stainless steel. In contrast, Karaca et al. (2015) reported that the phage ability to reduce biofilm formation was lower on plastic than on stainless steel. This is mainly because bacterial biofilms formed on different surfaces may differ according to the finish, roughness, hydrophobic interactions, physical and chemical stability, etc., of the material, which significantly interferes with the adhesion of cells and consequently with biofilm formation. For this reason, we investigated the activity of UPWr_S134 phage cocktail to combat *Salmonella in vitro* utilizing artificially infected plastic bell drinkers, commonly used in poultry farming. In this experiment, although the UPWr_S134 phage cocktail markedly reduced the number of *Salmonella* attached to the poultry drinker surface compared to controls, the level of *Salmonella* was relatively high and did not decrease during incubation. We assume that in such monospecies biofilms, the UPWr_S134 phage cocktail shows an inhibitory rather than a biocidal effect. This may be due to the fact that phage infection is affected by biofilms' defensive mechanisms such as inhibition of phage adsorption, penetration, and diffusion through biofilm structures to bacterial cells (Chang et al., 2022). Another reason is the proliferation of phage-resistant cells which naturally coexist with phage-susceptible cells (Simmons et al., 2020). To evaluate the hypothesis that the UPWr_S134 phage cocktail could be a useful tool in *Salmonella* elimination from the poultry drinkers in primary chicken production, we performed *in vivo* studies. In a novel infection model, we demonstrated for the first time that a *Salmonella*-targeted phage cocktail specifically and effectively eliminates *S*. Enteritidis from the abundant bacterial load located on the poultry drinker surface in the experimental chicken model. Interestingly, although *Salmonella* was present in drinking water, it was not detected in all biofilms. On the other hand, in the biofilm biomass containing *Salmonella*, the portion of this pathogen was modest. We assume that these results reflect the sheer number of bacteria in biofilms at the farm level as well as intraspecies interactions between them. To evaluate this hypothesis, further studies including the microbial community interactions within such biofilms are needed.

Poultry carcasses are primarily contaminated with pathogenic bacteria during slaughter, and these are transmitted to the carcass from infected internal organs and intestines (Golden et al., 2021). Even a small reduction in the number of pathogens has a great influence on public health. It was suggested by Powell et al. (2012) that a 2-log reduction in the number of *Campylobacter* on chicken carcasses would lead to a 30-fold drop in human cases of poisoning with contaminated chicken meat. Therefore, we investigated whether administration of UPWr_S134 phage cocktail with drinking water can also result in a decrease of *Salmonella* in internal chicken organs such as the spleen, the liver, and the bursa of Fabricius. In addition to the ability to eradicate *S*. Enteritidis from the surface of poultry drinkers, the phage cocktail UPWr_S134 significantly reduced the number of this pathogen in the bursa of Fabricius in phage-treated chickens. However, the reduction of *Salmonella* in the spleen and the liver was statistically insignificant in comparison to birds not treated with phages. Previously, similar results were obtained (Vaz et al., 2020). The authors reported that the incidence of *Salmonella* Enteritidis in the liver, spleen, and caecal tonsil samples did not significantly differ (*p* > 0.05) between phage-treated and untreated birds. The low ability of phages to reduce pathogens in internal organs may be the result of the method of bacteriophage administration to chickens. Meta-analytics suggested that the administration route may significantly impact phage treatment efficacy (Mosimann et al., 2021). Borie et al. (2008) reported that although a bacteriophage cocktail containing phages BP1, BP2, and BP3 significantly reduced the number of *S*. Enteritidis in birds treated with phages administered *via* coarse spray or drinking water, the latter method was less effective in the elimination of this pathogen from internal organs, partially due to phage inactivation attributed to stomach acidity. Therefore, the anti-*Salmonella* activity of the UPWr_S134 phage cocktail will be further investigated to develop the phage's applicability.

## Conclusion

In this study, we investigated phage ability to reduce *Salmonella* in poultry drinkers. We indicated the high potential of UPWr_S1, UPWr_S3, and UPWr_S4 bacteriophages and UPWr_S134 phage cocktail to reduce biofilm formed by *S*. Enteritidis *in vitro*. Our results revealed that UPWr_S134 phage cocktail treatment of artificially contaminated poultry drinkers resulted in a decreased number of *S*. Enteritidis attached to the drinker surface in laboratory conditions. Moreover, the study showed for the first time that the UPWr_S134 phage cocktail specifically and effectively eradicated *S*. Enteritidis from bacterial load located on the poultry drinker surface in an experimental chicken model, indicating that this phage cocktail is a promising candidate for *Salmonella* biocontrol at the farm level.

## Data Availability Statement

The original contributions presented in the study are included in the article/Supplementary Material, further inquiries can be directed to the corresponding author.

## Ethics Statement

The animal study was reviewed and approved by Institutional Local Ethical Commission for Animal Experiments at the Institute of Immunology and Experimental Therapy of the Polish Academy of Sciences in Wroclaw, Poland (protocol code 114/2015, 16.12.2015).

## Author Contributions

PK performed the study and drafted the figures. PŚ performed the statistical analysis. MK performed and supervised *in vivo* trials. AM provided assistance throughout the research. DM contributed to writing the manuscript. MK-B conceived and supervised the studies, had substantial inputs into the analysis and all drafts, obtained funding, and was a major contributor to writing the manuscript. All authors contributed to the article and approved the submitted version.

## Funding

This study was supported by the National Center for Research and Development, LIDER program no. LIDER/378/L-6/14/NCBR/2015. The APC was co-financed by Wroclaw University of Environmental and Life Sciences and University of Wroclaw.

## Conflict of Interest

The authors declare that the research was conducted in the absence of any commercial or financial relationships that could be construed as a potential conflict of interest.

## Publisher's Note

All claims expressed in this article are solely those of the authors and do not necessarily represent those of their affiliated organizations, or those of the publisher, the editors and the reviewers. Any product that may be evaluated in this article, or claim that may be made by its manufacturer, is not guaranteed or endorsed by the publisher.
