# Supplementary material for: Bacteriophage Cocktail Can Effectively Control Salmonella Biofilm in Poultry Housing
Source: Front Microbiol. 2022 Jun 29;13:901770. doi: 10.3389/fmicb.2022.901770 (PMC9277115; doi:10.3389/fmicb.2022.901770)
Supplement: Supplementary file 1 [file Data_Sheet_1.pdf]

I. 96-well microtiter plates

Table S1. One-way ANOVA and least significance difference (LSD) post-hoc comparisons of means of OD570 for *S.* Enteritidis ATCC 13076. Data presented as *p*-values between phages for each titer. Values depicted in red are statistically significant at a 95% confidence interval.

No.	Titer	Phage	UPWr_S1	UPWr_S3	UPWr_S4	UPWr_S1,3,4
1	10^9	UPWr_S1		0,086256	0,513132	0,902929
2	10^9	UPWr_S3	0,086256		0,239363	0,070962
3	10^9	UPWr_S4	0,513132	0,239363		0,441279
4	10^9	UPWr_S1,3,4	0,902929	0,070962	0,441279	
5	10^8	UPWr_S1		0,242039	0,375508	0,934095
6	10^8	UPWr_S3	0,242039		0,753550	0,214374
7	10^8	UPWr_S4	0,375508	0,753550		0,335924
8	10^8	UPWr_S1,3,4	0,934095	0,214374	0,335924	
9	10^7	UPWr_S1		0,101238	0,607672	0,650983
10	10^7	UPWr_S3	0,101238		0,224228	0,204433
11	10^7	UPWr_S4	0,607672	0,224228		0,950232
12	10^7	UPWr_S1,3,4	0,650983	0,204433	0,950232	
13	10^6	UPWr_S1		0,168195	0,707353	0,811391
14	10^6	UPWr_S3	0,168195		0,292780	0,240277
15	10^6	UPWr_S4	0,707353	0,292780		0,890245
16	10^6	UPWr_S1,3,4	0,811391	0,240277	0,890245	
17	10^5	UPWr_S1		0,233680	0,745655	0,630590
18	10^5	UPWr_S3	0,233680		0,143027	0,453249
19	10^5	UPWr_S4	0,745655	0,143027		0,427563
20	10^5	UPWr_S1,3,4	0,630590	0,453249	0,427563	
21	10^4	UPWr_S1		0,460945	0,827938	0,472207
22	10^4	UPWr_S3	0,460945		0,347042	0,984533
23	10^4	UPWr_S4	0,827938	0,347042		0,356246
24	10^4	UPWr_S1,3,4	0,472207	0,984533	0,356246	

Table S2. One-way ANOVA and least significance difference (LSD) post-hoc comparisons of means of OD570 for *S*. Enteritidis ATCC 13076. Data presented as *p*-values between titers for each phage. Values depicted in red are statistically significant at a 95% confidence interval.

No.	Phage	Titer	10^9	10^8	10^7	10^6	10^5	10^4
1	UPWr_S1	10^9		0,805567	0,493316	0,179186	0,137611	0,097546
2	UPWr_S1	10^8	0,805567		0,657278	0,262796	0,205297	0,148239
3	UPWr_S1	10^7	0,493316	0,657278		0,485293	0,393887	0,296984
4	UPWr_S1	10^6	0,179186	0,262796	0,485293		0,872226	0,717627
5	UPWr_S1	10^5	0,137611	0,205297	0,393887	0,872226		0,840264
6	UPWr_S1	10^4	0,097546	0,148239	0,296984	0,717627	0,840264	
7	UPWr_S1	10^9		0,698744	0,540019	0,152979	0,055294	0,006535
8	UPWr_S3	10^8	0,698744		0,818675	0,280820	0,109976	0,013644
9	UPWr_S3	10^7	0,540019	0,818675		0,388378	0,161599	0,021063
10	UPWr_S3	10^6	0,152979	0,280820	0,388378		0,561824	0,104209
11	UPWr_S3	10^5	0,055294	0,109976	0,161599	0,561824		0,268100
12	UPWr_S3	10^4	0,006535	0,013644	0,021063	0,104209	0,268100	
13	UPWr_S4	10^9		0,945231	0,023667	0,000160	0,000003	0,000001
14	UPWr_S4	10^8	0,945231		0,026927	0,000179	0,000003	0,000002
15	UPWr_S4	10^7	0,023667	0,026927		0,015693	0,000098	0,000048
16	UPWr_S4	10^6	0,000160	0,000179	0,015693		0,013482	0,005738
17	UPWr_S4	10^5	0,000003	0,000003	0,000098	0,013482		0,653621
18	UPWr_S4	10^4	0,000001	0,000002	0,000048	0,005738	0,653621	
19	UPWr_S1,3,4	10^9		0,355795	0,119624	0,000680	0,000822	0,000607
20	UPWr_S1,3,4	10^8	0,355795		0,488087	0,003798	0,004637	0,003369
21	UPWr_S1,3,4	10^7	0,119624	0,488087		0,014291	0,017474	0,012656
22	UPWr_S1,3,4	10^6	0,000680	0,003798	0,014291		0,915466	0,948897
23	UPWr_S1,3,4	10^5	0,000822	0,004637	0,017474	0,915466		0,864882
24	UPWr_S1,3,4	10^4	0,000607	0,003369	0,012656	0,948897	0,864882	

Table S3. One-way ANOVA and least significance difference (LSD) post-hoc comparisons of means of OD570 for *S.* Enteritidis 327 lux. Data presented as *p*-values between phages for each titer. Values depicted in red are statistically significant at a 95% confidence interval.

No.	Titer	Phage	UPWr_S1	UPWr_S3	UPWr_S4	UPWr_S1,3,4
1	10^9	UPWr_S1		0,875407	0,322164	0,613896
2	10^9	UPWr_S3	0,875407		0,397776	0,726014
3	10^9	UPWr_S4	0,322164	0,397776		0,610304
4	10^9	UPWr_S1,3,4	0,613896	0,726014	0,610304	
5	10^8	UPWr_S1		0,539852	0,061486	0,364040
6	10^8	UPWr_S3	0,539852		0,163789	0,755676
7	10^8	UPWr_S4	0,061486	0,163789		0,260426
8	10^8	UPWr_S1,3,4	0,364040	0,755676	0,260426	
9	10^7	UPWr_S1		0,678836	0,227429	0,881535
10	10^7	UPWr_S3	0,678836		0,120614	0,789746
11	10^7	UPWr_S4	0,227429	0,120614		0,182106
12	10^7	UPWr_S1,3,4	0,881535	0,789746	0,182106	
13	10^6	UPWr_S1		0,981631	0,106370	0,914054
14	10^6	UPWr_S3	0,981631		0,110305	0,895839
15	10^6	UPWr_S4	0,106370	0,110305		0,089629
16	10^6	UPWr_S1,3,4	0,914054	0,895839	0,089629	
17	10^5	UPWr_S1		0,765836	0,293565	0,823128
18	10^5	UPWr_S3	0,765836		0,438146	0,940381
19	10^5	UPWr_S4	0,293565	0,438146		0,397861
20	10^5	UPWr_S1,3,4	0,823128	0,940381	0,397861	
21	10^4	UPWr_S1		0,213919	0,493297	0,286470
22	10^4	UPWr_S3	0,213919		0,544808	0,840328
23	10^4	UPWr_S4	0,493297	0,544808		0,682589
24	10^4	UPWr_S1,3,4	0,286470	0,840328	0,682589	

Table S4. One-way ANOVA and least significance difference (LSD) post-hoc comparisons of means of OD570 for *S*. Enteritidis 327 lux. Data presented as *p*-values between titers for each phage. Values depicted in red are statistically significant at a 95% confidence interval.

No.	Phage	Titer	10^9	10^8	10^7	10^6	10^5	10^4
1	UPWr_S1	10^9		0,925935	0,224891	0,060912	0,010637	0,000065
2	UPWr_S1	10^8	0,925935		0,194417	0,051448	0,008917	0,000056
3	UPWr_S1	10^7	0,224891	0,194417		0,445774	0,107099	0,000523
4	UPWr_S1	10^6	0,060912	0,051448	0,445774		0,359207	0,002103
5	UPWr_S1	10^5	0,010637	0,008917	0,107099	0,359207		0,012180
6	UPWr_S1	10^4	0,000065	0,000056	0,000523	0,002103	0,012180	
7	UPWr_S3	10^9		0,762063	0,420490	0,066689	0,006138	0,001404
8	UPWr_S3	10^8	0,762063		0,609550	0,113580	0,010907	0,002453
9	UPWr_S3	10^7	0,420490	0,609550		0,259949	0,028777	0,006428
10	UPWr_S3	10^6	0,066689	0,113580	0,259949		0,217678	0,056507
11	UPWr_S3	10^5	0,006138	0,010907	0,028777	0,217678		0,434142
12	UPWr_S3	10^4	0,001404	0,002453	0,006428	0,056507	0,434142	
13	UPWr_S4	10^9		0,218607	0,045840	0,000361	0,000194	0,000031
14	UPWr_S4	10^8	0,218607		0,371205	0,003577	0,001811	0,000234
15	UPWr_S4	10^7	0,045840	0,371205		0,019988	0,009977	0,001151
16	UPWr_S4	10^6	0,000361	0,003577	0,019988		0,714597	0,145470
17	UPWr_S4	10^5	0,000194	0,001811	0,009977	0,714597		0,259942
18	UPWr_S4	10^4	0,000031	0,000234	0,001151	0,145470	0,259942	
19	UPWr_S1,3,4	10^9		0,844073	0,594203	0,274406	0,052853	0,010663
20	UPWr_S1,3,4	10^8	0,844073		0,735103	0,363627	0,075349	0,015489
21	UPWr_S1,3,4	10^7	0,594203	0,735103		0,560947	0,135477	0,029349
22	UPWr_S1,3,4	10^6	0,274406	0,363627	0,560947		0,335911	0,085379
23	UPWr_S1,3,4	10^5	0,052853	0,075349	0,135477	0,335911		0,400208
24	UPWr_S1,3,4	10^4	0,010663	0,015489	0,029349	0,085379	0,400208	

II. Stainless steel

Table S5. One-way ANOVA and least significance difference (LSD) post-hoc comparisons of means of OD570 for *S.* Enteritidis ATCC 13076. Data presented as *p*-values between phages for each titer. Values depicted in red are statistically significant at a 95% confidence interval.

No.	Titer	Phage	UPWr_S1	UPWr_S3	UPWr_S4	UPWr_S1,3,4
1	10^9	UPWr_S1		0,000002	0,000000	0,000030
2	10^9	UPWr_S3	0,000002		0,000000	0,006851
3	10^9	UPWr_S4	0,000000	0,000000		0,000000
4	10^9	UPWr_S1,3,4	0,000030	0,006851	0,000000	
5	10^8	UPWr_S1		0,005763	0,000061	0,006038
6	10^8	UPWr_S3	0,005763		0,000003	0,974955
7	10^8	UPWr_S4	0,000061	0,000003		0,000003
8	10^8	UPWr_S1,3,4	0,006038	0,974955	0,000003	
9	10^7	UPWr_S1		0,069567	0,000030	0,240232
10	10^7	UPWr_S3	0,069567		0,000006	0,433012
11	10^7	UPWr_S4	0,000030	0,000006		0,000011
12	10^7	UPWr_S1,3,4	0,240232	0,433012	0,000011	
13	10^6	UPWr_S1		0,773699	0,004883	0,772109
14	10^6	UPWr_S3	0,773699		0,007490	0,566974
15	10^6	UPWr_S4	0,004883	0,007490		0,003214
16	10^6	UPWr_S1,3,4	0,772109	0,566974	0,003214	
17	10^5	UPWr_S1		0,313829	0,006416	0,307202
18	10^5	UPWr_S3	0,313829		0,032431	0,062267
19	10^5	UPWr_S4	0,006416	0,032431		0,001447
20	10^5	UPWr_S1,3,4	0,307202	0,062267	0,001447	
21	10^4	UPWr_S1		0,080840	0,106193	0,028287
22	10^4	UPWr_S3	0,080840		0,863875	0,001604
23	10^4	UPWr_S4	0,106193	0,863875		0,002023
24	10^4	UPWr_S1,3,4	0,028287	0,001604	0,002023	

Table S6. One-way ANOVA and least significance difference (LSD) post-hoc comparisons of means of OD570 for *S*. Enteritidis ATCC 13076. Data presented as *p*-values between titers for each phage. Values depicted in red are statistically significant at a 95% confidence interval.

No.	Phage	Titer	10^9	10^8	10^7	10^6	10^5	10^4
1	UPWr_S1	10^9		0,011954	0,001099	0,000564	0,000042	0,000000
2	UPWr_S1	10^8	0,011954		0,216009	0,117149	0,006340	0,000014
3	UPWr_S1	10^7	0,001099	0,216009		0,708981	0,069386	0,000097
4	UPWr_S1	10^6	0,000564	0,117149	0,708981		0,132993	0,000180
5	UPWr_S1	10^5	0,000042	0,006340	0,069386	0,132993		0,002940
6	UPWr_S1	10^4	0,000000	0,000014	0,000097	0,000180	0,002940	
7	UPWr_S3	10^9		0,000007	0,000000	0,000000	0,000000	0,000000
8	UPWr_S3	10^8	0,000007		0,000124	0,000000	0,000000	0,000000
9	UPWr_S3	10^7	0,000000	0,000124		0,000339	0,000000	0,000000
10	UPWr_S3	10^6	0,000000	0,000000	0,000339		0,000083	0,000000
11	UPWr_S3	10^5	0,000000	0,000000	0,000000	0,000083		0,000000
12	UPWr_S3	10^4	0,000000	0,000000	0,000000	0,000000	0,000000	
13	UPWr_S4	10^9		0,328134	0,140774	0,055741	0,026271	0,020550
14	UPWr_S4	10^8	0,328134		0,587341	0,293521	0,155990	0,125488
15	UPWr_S4	10^7	0,140774	0,587341		0,598476	0,357915	0,297415
16	UPWr_S4	10^6	0,055741	0,293521	0,598476		0,685361	0,593473
17	UPWr_S4	10^5	0,026271	0,155990	0,357915	0,685361		0,896201
18	UPWr_S4	10^4	0,020550	0,125488	0,297415	0,593473	0,896201	
19	UPWr_S1,3,4	10^9		0,032766	0,000126	0,000042	0,000033	0,000011
20	UPWr_S1,3,4	10^8	0,032766		0,008625	0,002349	0,001734	0,000429
21	UPWr_S1,3,4	10^7	0,000126	0,008625		0,493299	0,398973	0,120425
22	UPWr_S1,3,4	10^6	0,000042	0,002349	0,493299		0,869415	0,353526
23	UPWr_S1,3,4	10^5	0,000033	0,001734	0,398973	0,869415		0,440837
24	UPWr_S1,3,4	10^4	0,000011	0,000429	0,120425	0,353526	0,440837	

Table S7. One-way ANOVA and least significance difference (LSD) post-hoc comparisons of means of OD570 for *S.* Enteritidis 327 lux. Data presented as *p*-values between phages for each titer. Values depicted in red are statistically significant at a 95% confidence interval.

No.	Titer	Phage	UPWr_S1	UPWr_S1	UPWr_S1	UPWr_S1
1	10^9	UPWr_S1		0,490551	0,471110	0,000946
2	10^9	UPWr_S1	0,490551		0,177428	0,002400
3	10^9	UPWr_S1	0,471110	0,177428		0,000386
4	10^9	UPWr_S1	0,000946	0,002400	0,000386	
5	10^8	UPWr_S1		0,020318	0,091118	0,000328
6	10^8	UPWr_S3	0,020318		0,362242	0,014642
7	10^8	UPWr_S4	0,091118	0,362242		0,003597
8	10^8	UPWr_S1,3,4	0,000328	0,014642	0,003597	
9	10^7	UPWr_S1		0,057400	0,725725	0,060173
10	10^7	UPWr_S3	0,057400		0,100830	0,976610
11	10^7	UPWr_S4	0,725725	0,100830		0,105617
12	10^7	UPWr_S1,3,4	0,060173	0,976610	0,105617	
13	10^6	UPWr_S1		0,102028	0,505966	0,382728
14	10^6	UPWr_S3	0,102028		0,283292	0,383045
15	10^6	UPWr_S4	0,505966	0,283292		0,825921
16	10^6	UPWr_S1,3,4	0,382728	0,383045	0,825921	
17	10^5	UPWr_S1		0,238979	0,856183	0,848917
18	10^5	UPWr_S3	0,238979		0,182540	0,313475
19	10^5	UPWr_S4	0,856183	0,182540		0,711025
20	10^5	UPWr_S1,3,4	0,848917	0,313475	0,711025	
21	10^4	UPWr_S1		0,210328	0,654649	0,799267
22	10^4	UPWr_S3	0,210328		0,395706	0,303726
23	10^4	UPWr_S4	0,654649	0,395706		0,845243
24	10^4	UPWr_S1,3,4	0,799267	0,303726	0,845243	

Table S8. One-way ANOVA and least significance difference (LSD) post-hoc comparisons of means of OD570 for *S.* Enteritidis 327 lux. Data presented as *p*-values between titers for each phage. Values depicted in red are statistically significant at a 95% confidence interval.

No.	Phage	Titer	10^9	10^8	10^7	10^6	10^5	10^4
1	UPWr_S1	10^9		0,214812	0,103587	0,047034	0,044008	0,016912
2	UPWr_S1	10^8	0,214812		0,659484	0,384194	0,365681	0,169467
3	UPWr_S1	10^7	0,103587	0,659484		0,659739	0,634091	0,332389
4	UPWr_S1	10^6	0,047034	0,384194	0,659739		0,971130	0,586663
5	UPWr_S1	10^5	0,044008	0,365681	0,634091	0,971130		0,611363
6	UPWr_S1	10^4	0,016912	0,169467	0,332389	0,586663	0,611363	
7	UPWr_S3	10^9		0,935639	0,900076	0,282493	0,114121	0,033454
8	UPWr_S3	10^8	0,935639		0,964232	0,317607	0,130873	0,038866
9	UPWr_S3	10^7	0,900076	0,964232		0,338452	0,141075	0,042224
10	UPWr_S3	10^6	0,282493	0,317607	0,338452		0,573475	0,226162
11	UPWr_S3	10^5	0,114121	0,130873	0,141075	0,573475		0,499028
12	UPWr_S3	10^4	0,033454	0,038866	0,042224	0,226162	0,499028	
13	UPWr_S4	10^9		0,956536	0,092971	0,055086	0,005246	0,005118
14	UPWr_S4	10^8	0,956536		0,084495	0,049875	0,004734	0,004619
15	UPWr_S4	10^7	0,092971	0,084495		0,769677	0,140686	0,137656
16	UPWr_S4	10^6	0,055086	0,049875	0,769677		0,225453	0,220948
17	UPWr_S4	10^5	0,005246	0,004734	0,140686	0,225453		0,989591
18	UPWr_S4	10^4	0,005118	0,004619	0,137656	0,220948	0,989591	
19	UPWr_S1,3,4	10^9		0,400043	0,000014	0,000000	0,000000	0,000000
20	UPWr_S1,3,4	10^8	0,400043		0,000050	0,000000	0,000000	0,000000
21	UPWr_S1,3,4	10^7	0,000014	0,000050		0,000098	0,000004	0,000000
22	UPWr_S1,3,4	10^6	0,000000	0,000000	0,000098		0,040082	0,001583
23	UPWr_S1,3,4	10^5	0,000000	0,000000	0,000004	0,040082		0,104194
24	UPWr_S1,3,4	10^4	0,000000	0,000000	0,000000	0,001583	0,104194	
